# Research performance and age explain less than half of the gender pay gap in New Zealand universities

**DOI:** 10.1371/journal.pone.0226392

**Published:** 2020-01-22

**Authors:** Ann Brower, Alex James

**Affiliations:** 1 School of Earth and Environment, University of Canterbury, Christchurch, New Zealand; 2 Te Pūnaha Matatini Centre of Research Excellence, Auckland, New Zealand; 3 School of Mathematics and Statistics, University of Canterbury, Christchurch, New Zealand; University of Edinburgh, UNITED KINGDOM

## Abstract

We use a globally unique dataset that scores every individual academic’s holistic research performance in New Zealand to test several common explanations for the gender pay gap in universities. We find a man’s odds of being ranked professor or associate professor are more than double a woman’s with similar recent research score, age, field, and university. We observe a lifetime gender pay gap of ~NZ$400,000, of which research score and age explain less than half. Our ability to examine the full spectrum of research performance allows us to reject the ‘male variability hypothesis’ theory that the preponderance of men amongst the ‘superstars’ explains the lifetime performance pay gap observed. Indeed women whose research career trajectories resemble men’s still get paid less than men. From 2003–12, women at many ranks improved their research scores by more than men, but moved up the academic ranks more slowly. We offer some possible explanations for our findings, and show that the gender gap in universities will never disappear in most academic fields if current hiring practices persist.

## Introduction

Many have noticed that fewer women make it to the top ranks of academia[1–3], and have posited a gender gap in both rank and pay. Some have explained this gap by: men in universities are older and publish more[4–14]; women are more likely to take family-related career breaks[2] and less likely to apply for promotion or jobs elsewhere[15]; and sexism[16], politely renamed unconscious bias[17].

Recent studies of the dearth of women at top ranks outside academia have re-examined the common explanations, of ‘women don’t ask’ for promotions[18], and strive for less[19]. Recent studies have found that women do ask for promotion[20] at similar rates to men[21], but are less likely to get promoted[20]. Further, young women are as ambitious as men; but perceived inequities in advancement opportunities curb women’s ambitions more than having children does[22].

Within academia, studies have examined another common explanation, called ‘demographic inertia’, in which the current preponderance of older men at the top pay grades is a hold-over from bygone eras of male-dominated universities that will fade with time[23,24]. Yet new evidence (ours included) shows gender balances in academic staff are changing too slowly to achieve gender parity[25,26], suggesting there is more than just inertia at play. Indeed women are promoted more slowly through academia[27]. In Japan, parental and marital status help explain gender differences in promotion rates[27], while in Scandinavia they do not [28].

A plethora of studies has observed many other types of gender differences in academia[3,4,24,29–54]. Beyond gendered gaps in rank, promotion, and pay, studies suggest women are disadvantaged in peer review[55–57], research funding[49,58,59], authorship ranking[11], citation rates in high impact journals[60], administrative service[61–64], and teaching demands[65] and teaching evaluations[66–68]. Pregnant women[69] and mothers[70,71] appear further disadvantaged in employment both inside[2] and out of the university. There is even evidence of bias against research that finds gender bias[72,73].

Our study stands alone in the global literature about academic gender biases and pay gaps, which are easier to posit than to quantify[13,74–76]. While other studies have extrapolated research performance from bibliometrics[5], our data scores individual research performance on a fine scale (from 0 to 700) for every university academic in New Zealand (NZ). Where previous studies have examined a field or department [8,9,15,53,58,77], ours covers all academics in all fields at all universities in New Zealand. Where others have used surveys or extrapolation to estimate salary[53], we have a salary band for every individual in our study because NZ universities follow a clear pay scale available for all institutions (S1 Fig). Although some NZ academics negotiate their own salary off the scale, all academics apply for promotion through academic ranks similarly. This renders possible gender differences in negotiation dynamics[15] less relevant in NZ than in other countries.

We use this globally unique dataset that scores individual research performance for every New Zealand academic to ask whether there is a gender pay gap in NZ universities, and to decipher whether and how research performance explains it.

### New Zealand’s performance based research fund

New Zealand’s unique Performance Based Research Fund (PBRF) scores each individual’s research performance in a holistic and nuanced way to include peer esteem and research contributions, in addition to publications (see Section 1 S1 File)[78,79]. Primarily, PBRF is a tool to distribute a pool of government research funds amongst public institutes of tertiary education. However, beyond institutional funds allocation, PBRF aims to foster an environment that promotes quality research and ensures that teaching is grounded in research findings[80].

PBRF’s comprehensive scoring of each individual allows comparisons across departments and institutes for allocation purposes, and across individuals for our research. Scores are calibrated within and across academic fields, and clustered into grades: 600–700 A; 400–599 B; 200–399 C; 0–199 R (Research inactive). Grades are strictly confidential; only the individual, his or her faculty dean or college Pro-Vice Chancellor (not head of department or school), and the Vice Chancellor (chief executive of a NZ university) and his or her Deputy know who got what grade.

PBRF evaluates a research portfolio for each academic researcher in all public tertiary education institutes. There are tertiary institute staff who do not identify as researchers, particularly at polytechnics, and do not submit a portfolio; but the vast majority of university staff submit portfolios. Thus, in universities, academic researchers comprise a slightly smaller group than academic staff. We include only university researchers in our study, not researchers from polytechnics.

PBRF panels have reviewed and scored a detailed research evidence portfolio for every academic researcher in the country’s eight universities (7,587 portfolios; 5,844 unique individuals) three times over nine years (2003, 2006, 2012). At least two panel members evaluated and scored each portfolio (see sections 1, 3 S1 File).

Globally, PBRF is the only nationally comprehensive research evaluation scheme that assesses all individual academic researchers in all fields of study, across all academic institutes in the country with the same metric [80]. Other countries, such as the United Kingdom, South Africa, and Australia, assess research excellence of departments or institutions, but not at the individual level[80]. Canada and the US also have some national research assessments, but these are restricted to medical fields[80].

Evaluation of the evidence portfolios is done by 42 groups of 2–4 external peer reviewers, clustered into panels by subject area (e.g. Cell and Molecular Biology; Earth sciences; Political Science, International Relations, and Public Policy), as well as expert advisors. To protect anonymity, we have clustered the 42 areas into 6 fields of study (Science; Engineering; Commerce and Law; Medicine; Arts; Education). There is also a moderation panel to ensure consistency across disciplinary panels, resolve inconsistencies, and advise the Tertiary Education Commission (TEC, the government agency that oversees tertiary education and the PBRF) about consistency issues[81].

PBRF assessment emphasises quality and impact over quantity. In addition to publishing articles, PBRF research excellence includes: leading-edge knowledge, its application, public dissemination, national or global impact, and post-graduate supervision[80]. To assess a portfolio, PBRF reviewers examine impact and contribution statements of each researcher’s top 4 research outputs (e.g. books, journal articles, art exhibitions) and the outputs themselves, of the individual’s own choosing and description. The assessment panel also evaluates a list of the individual’s next best 20 outputs. In 2012, research outputs constituted 70% of an individual’s PBRF score. The remaining 30% assessed self-described accounts of peer esteem (e.g. research awards, invitations to give key note addresses) and contributions to the research environment (e.g. journal editorship, conference organisation).

## Results

### The academic gender rank gap

#### Men’s odds of being ranked associate or full professor are over double women’s odds

In New Zealand, women’s odds of being ranked, and paid, as Professor or Associate Professor, (i.e. in the professoriate) are lower than men’s. In 2012, 43.5% of men (of 2,737) and 21.1% of women (of 1,739) were ranked Associate Professor (AP) or Professor (P), yielding a significant gender odds ratio of being AP or P (OR, men:women) of 2.9. In 2003, the gender odds ratio was 3.76 (Fisher test 95% confidence intervals 2012: [2.51, 3.31], *p* = 10^-55^; 2003: [3.04, 4.64], *p* = 10^-41^). However, women have lower research scores (S2 Fig) and the average woman is 1.78 years younger than the average man.

We first ask whether research score and age explain the observed gender odds ratios, which translate to a gender gap in academic rank (for Methods see section 4, S1 File).

#### Research score, subject area, and age reduce, but do not explain away, the gender odds ratios

**Fig 1:**
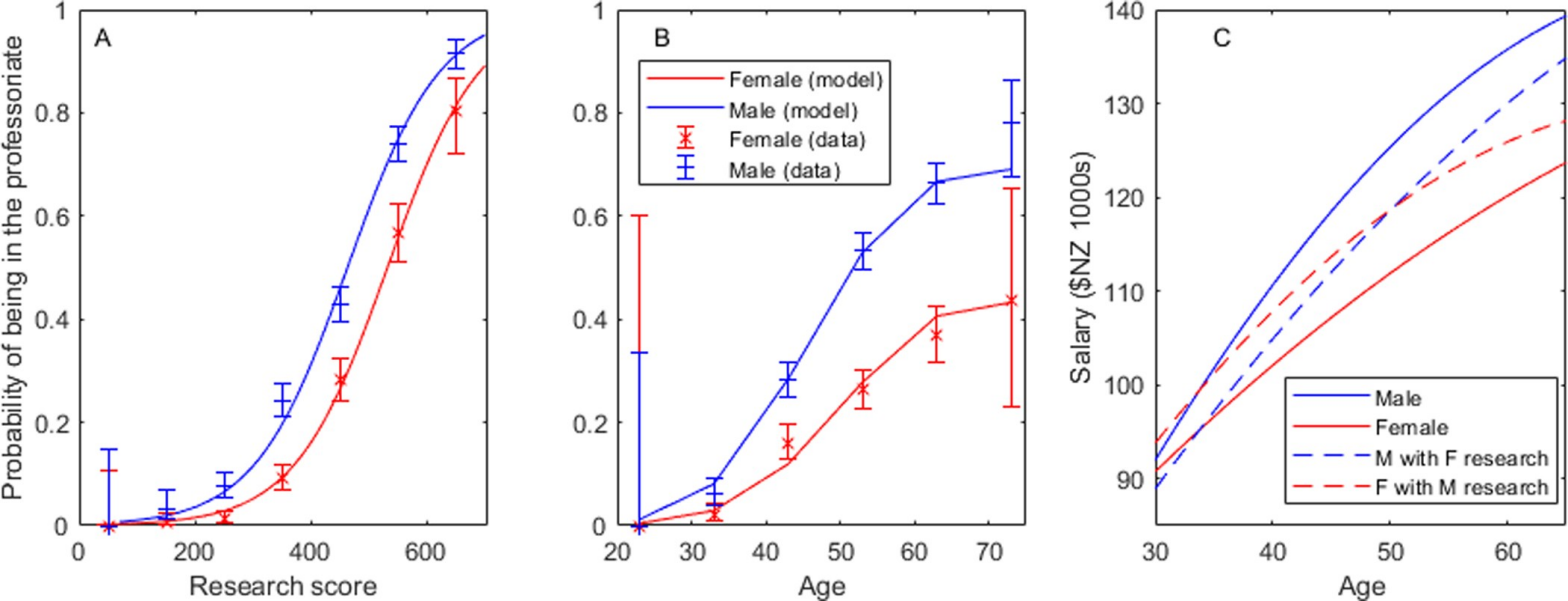
Accounting for recent research score or age, the probability of being ranked professor or associate professor is always higher for a man than for a woman. Even when women match the research scores of men, they are paid less.

Neither controlling separately for recent research performance with the 2012 research score, nor age using logistic regression (S2 Table, section 4 S1 File) diminishes the gender odds ratio of being in the professoriate (Score: OR = 2.36, *p* = 10^-24^; Age: OR = 2.93, *p* = 10^-45^) (Fig 1A and 1B). Controlling for gender, age, 2012 research score, research field, and university together only decreases the gender odds ratio of being in the professoriate to 2.2 (*p* = 10^-13^). A woman’s odds of being a full Professor, rather than AP or P, are lower still (OR = 2.8. *p* = 10^-14^) (S2 Table).

Breaking field into 42 subject areas shows variability amongst areas (section 7, S1 File). When predicting the probability of being in the professoriate, most have a gender odds ratio above 2; in only 9 subject areas are women advantaged (i.e. have an odds ratio less than 1) (S5 Table). This variability neither drives the gender odds ratios, nor explains the observed gender rank gap.

To look for generational differences, we examine only researchers below age 50. Within this cohort, controlling for score, field, and university decreases the gender odds ratio of being in the professoriate to 1.5; but it is still significant (*p* = 0.02) (S2 Table). An equivalent analysis of the 2003 sample shows slightly higher gender odds ratios (S2 Table).

#### Neither superstars nor the male variability hypothesis explain away the gender rank gap

Next we examine the ‘superstars’ at the top end of the research score spectrum. The ‘male variability hypothesis’ of evolutionary psychology claims men are over-represented in the top and bottom tails of population distributions, with women clustered in the middle [82]. In our first approach, we restrict the 2012 sample to only A-grade researchers. The over-representation of men in the top tail seems to support the hypothesis, but female A-grade researchers are still significantly less likely to be ranked at AP or P than male A-grades (OR = 2.1, *p* = 0.06) (S2 Table). This suggests male dominance in the top tail of research does not explain male dominance of the top academic ranks.

An alternative approach includes research score-squared in the original analysis, allowing for a disproportionate reward for high performance. However, score squared yields no significance (*p* = 0.25), a vanishingly small coefficient (4 × 10^-6^), and has almost no effect on the gender odds ratio of 2.2 (*p* = 10^-13^) (section 4, S1 File).

Together, these approaches show our findings are robust at the top tail, and not explained by male variability. Although the preponderance of superstars are male, they neither influence nor explain the observed gender odds ratio.

### The academic gender pay gap

#### There is an academic gender pay gap; and research performance, age, and field explain less than half of it

Next we examine academic pay. We ask whether a gender pay gap exists, and how much of it is explained by age, research score, field, and other observable variables. We first use research scores and published salaries by rank (S1 Fig and S1 Table) to predict the salary of an average performing man and woman, following his or her expected lifetime trajectory of research scores in each academic field and university (Table 1) (section 5, S1 File).

**Table 1:** Between 30% and 60% of the academic gender pay gap is not attributable to research performance. Expected lifetime earnings across the six different academic fields for men and women with an average research output. Earnings for individuals following the expected research trajectory of the opposite sex. Proportion of the pay gap which is not attributable to research performance difference (see S1 File, section 5).

	Expected highest research score			Lifetime earnings in $NZ 1000s				
Field	Male	Female	Final salary diff in $NZ 1000s	Male	Female	F with M research	Pay gap attributed to score	Gender performance pay gap
					(Diff to male)	(Diff to male)		
Arts	426	421	8.8	3965	3810	3868	62.3%	37.4%
					(-155)	(-97)		
Science	474	433	15.6	4312	3915	4118	48.8%	51.2%
					(-397)	(-194)		
Business	405	383	12.7	4224	3935	4047	61.3%	38.7%
					(-289)	(-177)		
Engineering	454	430	10.7	4229	4005	4136	41.7%	58.3%
					(-224)	(-93)		
Medicine	421	366	24.2	5002	4309	4531	67.9%	32.1%
					(-693)	(-471)		
Education	374	342	11.6	3878	3634	3766	46%	54.0%
					(-244)	(-112)		

For example, Fig 1C (solid lines) predicts the lifetime earnings of an average man and woman in Science at the University of Canterbury. By retirement at 65, our average female scientist would have a salary of $15,600 less than our average man (Table 1). Through her career (aged 30–65), she will earn $397,000 less than him–about 80% of the 2018 median house price in Christchurch, their home city. She would need to work three additional years at her highest salary to match his lifetime earnings. A woman who follows the higher, average male expected research trajectory (Fig 1C, dashed red line) will earn $194,000 less, over her career, than a man on the same research trajectory–about 40% of a house.

In Science, 49% of the observed gender pay gap is explained by women’s expected lower research outputs, i.e. a woman on the male research trajectory has only 51% of the expected pay gap. We call this 51% a gender performance pay gap. Medicine has the highest gender pay gap (Table 1) and the majority of this gap can be explained by women’s lower research scores leaving only 32% in the unexplained gender performance pay gap. Conversely, the pay gap in Engineering is much lower; but increasing a woman’s research score to match that of a man still leaves 58% of the gap unexplained. There are a number of variables not observed in this study that could contribute to the gender performance pay gap; we discuss them in the Discussion section below.

#### If men and women improve their scores similarly, they are not promoted similarly

We also explore promotion and salary increase patterns over time with the sub-sample of individuals who participated in both the 2003 and 2012 PBRF scoring exercises. First we consider only those at the lower ranks (L and SL) in 2003 (Table 2). After controlling for field and age, women in this group improved their research score by 13 points more than men on average (*p* = 0.033). Yet men in this group had higher odds of promotion to AP or P (Promotion rates: men 46%, women 34%; OR = 1.8, *p* = 1.4 × 10^-5^, controlling for 2012 research score, age squared, and field, see S1 File section 6). Men in this group also received a higher pay rise over the period ($1,249 more per year), but this was not significant (*p* = 0.14).

**Table 2:** Even when women improve their research more than men, they are less likely to be promoted. The promotion chances and salary improvements of men and women between 2003 and 2012 split by 2003 rank. Positive score and salary differences imply men improved by more than women. The cohort is then split further by minimum rank reached by 2012, giving the probability of reaching at least this rank and the gender odds ratio (OR) and p value (p-val). Columns marked * are the gender coefficient of linear models accounting for other variables and associated coefficient p value (see section 6, S1 File).

Rank 2003	N	Mean Score 2003	Mean Score 2012	Improvement 2003–2012		Rank (2012)					
				*Score diff	*Salary diff	SL/AP/P		AP/P		P	
				(p-val)	(p-val)	Promoted	*OR (p-val)	Promoted	*OR	Promoted	*OR
									(p-val)		(p-val)
L/SL(F)	501	299.4	412.2	-13.2	1249.2			34.10%	1.8		
L/SL(M)	775	348	420.9	(0.033)	(0.144)			46.20%	(0.000)		
L (F)	209	246.3	389	6.1	3304.9	83.70%	1.9 (0.078)	12.40%	1.8	0.50%	8.9
L (M)	218	291.6	412.7	(0.578)	(0.009)	90.80%		20.20%	(0.086)	5.00%	(0.067)
SL (F)	292	337.4	428.8	-20.8	2384.5			49.70%	1.6	13.00%	1.5
SL (M)	557	370.1	424.1	(0.006)	(0.020)			56.40%	(0.027)	15.60%	(0.138)
AP (F)	45	457.9	508.4	-7.9	2171.9					62.20%	1.5
AP (M)	193	467.1	494.4	(0.647)	(0.307)					67.40%	(0.349)

Next we examine each rank and promotion separately (Table 2, section 6 S1 File). The most significant differences are in the promotions from Senior Lecturer where, after controlling for age and field, women improved their scores by more but had lower salary rises and promotion odds than men. Female SLs improved their scores by almost 21 points more than male SLs (*p* = 0.006), more than at any other rank. Yet male Senior Lecturers had significantly higher odds of being promoted to AP or P (*OR* = 1.6; *p* = 0.0027). The gender difference in pay rise per year was $2384.5 (*p* = 0.02). Corresponding results are seen at the Lecturer level where the gender difference in score improvement is negligible, but men’s pay rises are higher. Only at the highest promotion level, from AP to P, are all the gender differences insignificant (though even here, men’s odds of promotion are higher).

### Modelling gender equity in the future university

**Fig 2:**
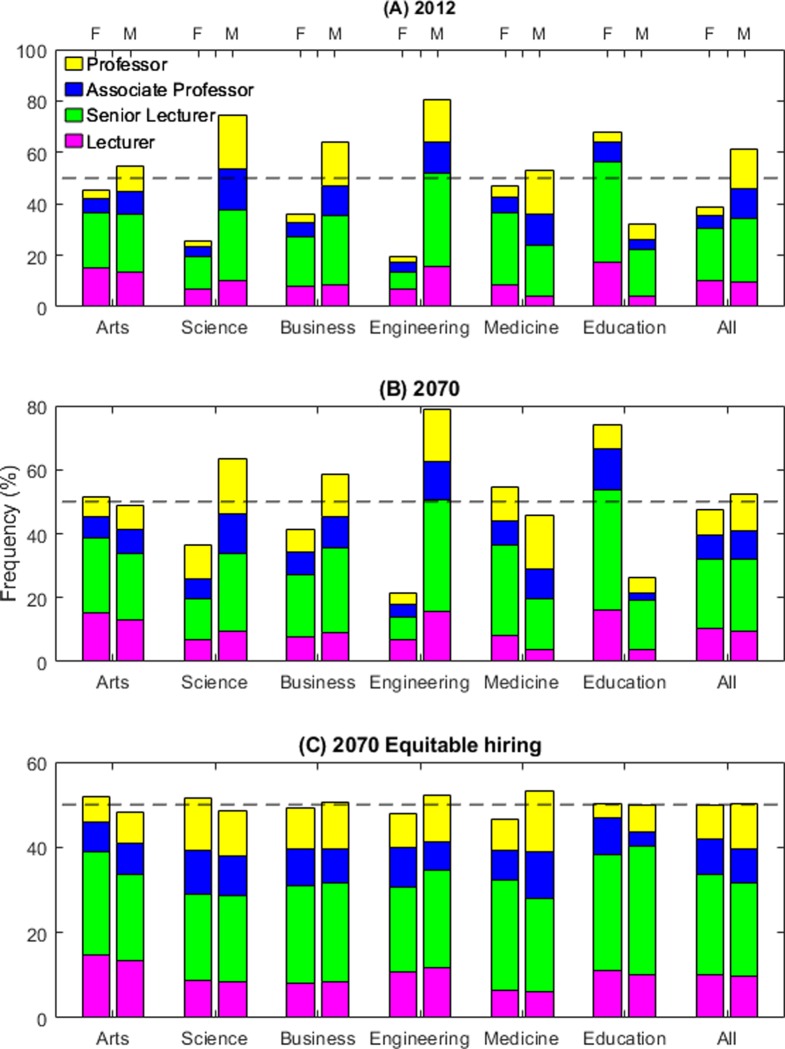
Under current hiring practices few fields will reach gender parity. (A) The 2012 rank distribution by gender of each field. (B) The projected rank distribution in 2070 (equilibrium). Despite the overall gender balance being close to parity, men are still more likely to be at the higher ranks and individual fields show large differences. (C) With fully equitable hiring policies, the differences are smaller but women are still more likely to be at a lower rank.

Finally, we create a transition model with Leslie matrices to envisage the future university (section 8, S1 File). Fig 2B shows current hiring practices are leading to a more equitable distribution for the entire population, but there will continue to be more men both overall and at higher ranks. In Science, Engineering, and Business, men will similarly continue to dominate. By contrast, in Medicine, Education, and Arts, women will constitute the majority of staff, while men will dominate the professoriate. If we move to gender parity in hiring, in which new hires at each rank are equally likely to be male or female (section 8, S1 File), most fields will approach gender parity both overall and within ranks (Fig 2C). But no field will reach gender parity. This suggests hiring, promotion, and attrition patterns all contribute to the preponderance of men at universities’ top ranks.

## Discussion

We used a globally unique dataset that scores research performance for every academic researcher in a country on a single metric to try to explore the observed gender pay gap in universities. Observable data, including research score, age, subject area, and university, explain less than half.

### Unobserved factors and the “double-whammy”

There are several possible explanations for, and caveats to, our finding that men occupy higher ranks, and earn more, than women with the same research score. Our nationwide data eliminate research and age as explanations for the gender pay gap, but cannot measure the other components of academic advancement–teaching and service to the university, community, or discipline.

International literature suggests women teach[65] and serve more[61–63], casting doubt on less or worse teaching or service explaining the observed gender pay gap. However, students and organisations expect more from women[61–63,65,77], and women are disadvantaged in teaching evaluations[66,68]. This suggests a “double-whammy effect”, in which universities over-demand and under-reward women’s teaching and service, might explain our findings in part. Women’s research scores are lower, suggesting they might suffer doubly in promotions from simultaneously researching less due to higher teaching and service expectations, while still failing to meet the burden of those higher expectations.

### Hiring patterns and research quantity vs. quality

It is possible our results reflect a pattern in which men are hired at higher steps within the ranks, then promoted at similar speeds. We know neither which step within the lecturer and senior lecturer ranks each individual occupied in 2003, nor exact promotion timings (S1 Fig).

It might also be that the PBRF scoring favors women, with its emphasis on quality and impact instead of quantity. International research suggests that men publish more, but the impact of each output is similar[6]. If promotions favor quantity while PBRF favors impact, men might progress more quickly than PBRF scores would predict, explaining part of the observed gender performance pay gap.

However both international findings—that evaluation exercises often favor men [33,68]—and our own findings—that women score lower on PBRF (Fig 1)—render this explanation unlikely. If PBRF favors men, our findings will underestimate the gender pay gap. A bibliometric study of PBRF could contribute to answering questions of gender bias within PBRF.

## Conclusion

Our dataset reflects a nation-wide study of almost 6,000 individuals and their positions within academia. Taken singly, the internal logic of each hiring or promotion decision might cohere. But taken together, they reveal a strong pattern in which a man’s odds of being ranked associate or full professor are more than double those of a woman with equivalent recent research score and age.

Indeed research score and age explain less than half of the approximately $400,000 lifetime gender pay gap in NZ universities. Although equity policies in hiring and promotions will narrow the gender gap over time, the ivory tower’s glass ceiling remains intact.

## Supporting information

S1 File Supporting information for Brower and James 2019. (DOCX)

S1 Fig University of Canterbury’s academic rank and salary steps, according to the collective employment agreement (2019–2021) negotiated between the university and the tertiary education union (from https://www.canterbury.ac.nz/hr/ea/rs_cea01.pdf). This scale is similar in all New Zealand universities, though salaries differ.
(TIF)

S2 Fig In 2012, women were more likely than men to be lecturers, to be older at lower ranks, and to have a lower research grade. (A) The frequency of each academic rank split by gender. (B) The frequency of each research grade. (C) The expected age of the individuals at each rank. (D) The expected age of the individuals at each research grade.
(TIF)

S1 Table Rank to salary conversion used for each institution. **Taken from 2018 salaries in academic collective agreements available from** www.teu.ac.nz.
(XLSX)

S2 Table All possible logistic regression models to predict the probability of being in the professoriate (AP or P) in 2012 or 2003 separately. The table shows all regression models used, the gender coefficient (if included), associated p-value and corresponding odds ratio. Using the entire 2012 (or 2003) dataset the best fit model, as predicted by AIC, area under the receiver-operator curve or percentage of correct predictions, contains the gender variable. When a subset of the data is used (e.g. only A-grade researchers; only those under 50), or we predict the probability of being a full professor, gender is still a significant predictor variable in the best fit models.
(XLSX)

S3 Table All possible linear regression models to predict an individual’s salary and PBRF score in 2012. The table shows all regression models used, the gender coefficient (if included), associated p-value and corresponding odds ratio. Note that we used interactions between gender and other terms in these models, rendering the gender coefficient less explanatory in this case. The sample is the same as that in S2 Table. The sample size of men and women is given. For the salary model, the top four models showed almost no difference by AIC so the most parsimonious, i.e. the model with the least interactions, was chosen. Choosing one of the other models gave a slight quantitative change to Table 2 but did not change the overall results.
(XLSX)

S4 Table Promotion rates, hiring probabilities, and leaving rates for each field, as used in the Leslie matrix transition model. (XLSX)

S5 Table The probability of being in the professoriate using all 42 subject areas. (XLSX)
