# Supplementary material for: Research performance and age explain less than half of the gender pay gap in New Zealand universities
Source: PLoS One. 2020 Jan 22;15(1):e0226392. doi: 10.1371/journal.pone.0226392 (PMC6975525; doi:10.1371/journal.pone.0226392)
Supplement: S1 File — (DOCX) [file pone.0226392.s001.docx]

**S1 File. Supporting information for Brower and James 2019**

**This file includes:**

Materials and Methods

1. The Performance Based Research Fund
2. NZ academic ranks promotions and salaries
3. Data
4. Statistical methods
5. Modelling salary
6. Exploring subject area
7. Modelling the future university

Captions for Figures S1 Fig, S2 Fig

Captions for Tables S1 Table, S2 Table, S3 Table, S4 Table, S5 Table (attached in separate Excel files)

Materials and Methods

1. The Performance Based Research Fund (PBRF)

New Zealand’s Performance Based Research Fund’s assessment is unique in the world. It scores every academic’s research performance from 0-700 points. Other countries such as Australia and the UK score research performance (and allocate research funds) in aggregate, by department or university. But New Zealand is the only country to assess a research portfolio for every academic in the country.

A portfolio contains full texts and impact summaries of an academic’s nominated four best publications (books, articles, exhibitions, etc), a list of additional publications and postgraduate supervisions, and evidence of the academic’s contribution to the research environment and peer esteem (journal editorial posts, speaking invitations, awards, etc.) during the assessment period. PBRF takes part-time employment, and special circumstances (illness, heavy administrative load, etc) into account.

PBRF panels consist of national and international experts grouped by research field. In 2012, the panels reviewed their preliminary scores for evidence of patterns of bias before moderating and finalizing the scores[1](at page 137). However it was only the latest round (2018, data not yet available for inclusion in this study) that panelists did received unconscious bias training. Portfolios also contain data not used in the assessment, on gender, age, academic rank, and university(1–5).

In 2012, PBRF gave each individual academic three component scores, for research outputs, peer esteem, and contribution to the research environment. These were combined in the ratio 70:15:15 to give an overall score from 0 to 700.

Scores were then clustered into grades (600-700=A; 400-599=B; 200-399=C; 0-200=R (research inactive)). An individual who began doing independent (post-degree) academic research within the assessment period may be classified as new and emerging if they are in either the R or C grades, becoming R(NE) or C(NE).

1. New Zealand academic ranks, promotions, and salaries

New Zealand universities rank academic staff similarly to the UK and Australia: lecturer (L), senior lecturer (SL), associate professor (AP) and professor (P). Within each rank, there are between four and eight salary steps, which are not recorded by PBRF (S1 Fig).

**S1 Fig**: University of Canterbury’s academic rank and salary steps, according to the Collective Employment Agreement (2019-2021) negotiated between the university and the Tertiary Education Union (from <https://www.canterbury.ac.nz/hr/ea/rs_cea01.pdf>). This scale is similar in all New Zealand universities, though salaries differ.

All New Zealand universities follow a similar promotion structure. Starting academics are usually hired at the lecturer level which contains approximately 6 pay steps. Progression through this rank is automatic. Individuals move up a pay step at the end of each year, but can apply for accelerated progression of two or more steps.

At some point, individuals apply to move to the senior lecturer (SL) rank. Progression through this rank is also automatic each year for about 5 steps. At this point, individuals reach a ‘bar’ and must apply for promotion to the upper half of the SL scale which consists of three steps. A Senior Lecturer above the bar is equivalent to a US-based Associate Professor.

The next step is Associate Professor (AP, equivalent to Reader in the UK). Past the first step of AP, progression to higher steps is not automatic and usually only happens upon successful application. The highest rank is Professor (P). It is possible for individuals to take large steps through the scale; for example it is not unheard of for individuals to move straight from senior lecturer to professor.

Promotions applications are usually decided by a university committee, which considers all applications annually. Applications are judged on research, teaching, and service to the university and the discipline. New hire, mid- to late-career academics may be appointed at any appropriate point in the ranking system.

We assigned salaries to ranks at each university separately using the 2018 salaries from the academic collective agreement ([www.teu.ac.nz](http://www.teu.ac.nz)) for each university (S1 Table). In addition, the University of Otago has a separate pay scale for Medicine, which was used. To assign salary, we used the mean value of the upper and lower limit for each rank. At some universities, upper salaries for the higher ranks are not given. In these cases, a conservative estimate that aligned with ranges from other institutions was used. In practice, it is likely these upper limits are higher than our estimates here. This would increase the size of the gender pay gap, as the men predominate in the upper ranks.

Age and research performance are important factors in academic rank. S2 Fig shows the summary statistics of the PBRF dataset split by rank, grade, and gender. In 2012 women’s research scores overall are lower, more women receive R or C grades, and under half as many receive A grades (men 16.0%, women 7.2%) (S2 Fig). Overall, men are 1.7 years older, but by rank only male professors are older, women are older at all other ranks (two-sided t-tests, L: 2.1 years, $p=0.002;$SL: 0.73 years, $p=0.09$; AP: 0.11 years, $p=0.87$; P: -1.3 years, $p=0.103$, Overall: -1.7 years, $p={10}^{-7}$). By contrast, women are younger than men at all active research grades (two-sided t-tests, A: 2.8 years, $p=0.006$, B: 1.2 years, $p=0.02$, C: 0.95 years, $p=0.08$). There is no significant age difference in the research inactive and new and emerging categories. Together, these suggest women are hired at lower ranks or spend longer there, but are not older when hired.

**S2 Fig: In 2012, women were more likely than men to be lecturers, to be older at lower ranks, and to have a lower research grade.** (A) The frequency of each academic rank split by gender. (B) The frequency of each research grade. (C) The expected age of the individuals at each rank. (D) The expected age of the individuals at each research grade.

1. Data

The data were analyzed anonymously, under a non-disclosure agreement with the New Zealand Tertiary Education Commission, the owner of the dataset.

The original PBRF dataset contained 21,051 portfolios from 11,549 individuals recorded at three time points 2003, 2006, 2012. We did not use the 2006 data in this study because the 2006 round was not compulsory, so individuals could self-select in or out.

We did not include individuals at a College of Education because the colleges were added to universities after the advent of PBRF, so research activity might follow a different pattern. Note that there are still individuals researching in the field of Education who were based at Universities.

Where gender or age were not stated, appropriate data for that person from a different time point was used if possible. There were no explicit records of transgender individuals, though these may have been recorded as gender not stated. Individuals for whom either gender or age could not be determined were excluded from the study. Date of birth was recorded by decade to protect anonymity, so the mean value for age was used.

Individuals with an academic rank outside the usual Lecturer, Senior lecturer, Associate professor, or Professor scale were not included in the study. Excluded individuals included those on research only positions (which in the NZ system are often fixed-term), teaching only positions, and senior management. Portfolios with a final score of zero and no component scores were also excluded.

1. Statistical methods

All regressions were carried out in Matlab 2017b using the fitglm function. Models that showed errors in fitting due to small sample sizes in some categories were excluded. Unless explicitly stated, interactions between terms were not included. Interactions were rarely significant and did not improve model performance as measured by either AIC or area under the receiver-operator curve (ROC). The odds ratio for gender is calculated as the exponential of the gender coefficient in the logistic regression. In linear regressions, the coefficient for gender is quoted as is. All p-values quoted are for the coefficient, not the overall model. Finally, we present the proportion of correct predictions (Correct (%)).

S2 Table shows the outputs of all candidate models tested to predict the probability of being in the professoriate. When the model is applied to all individuals as a binary single predictor variable Gender often has surprisingly good predictive power (e.g. in 2012 $\boldsymbol{ROC=0.617}$, correct in 61.4% of cases). The other variables combined give a much improved prediction. However, when added to any model Gender is a significant predictor variable ($\boldsymbol{p<}\boldsymbol{10}^{\boldsymbol{-10}}$) with an odds ratio in favour of men and the AIC score is always improved. For example, when we compare the best model

$$\boldsymbol{P}\left( \boldsymbol{being in the professoriate} \right)\boldsymbol{\sim Gender+Research score+Ag}\boldsymbol{e}^{\boldsymbol{2}}\boldsymbol{+Field+Inst}$$

with the second best, i.e. the same model without the Gender coefficient, the AIC coefficients are different by more than 50 showing a significant model improvement despite the very small improvement in ROC and percentage of correct predictions.

Similar results are achieved when the analysis is carried out on subsections of the data, i.e. individuals with a grades only or under 50s. We also modify the analysis to predict the probability of being a full professor rather than an Associate or Full professor, again the results have the same overall conclusions.

A separate model including score-squared to test the “male variability hypothesis” did not explain the preponderance of men in the professoriate. The model output was:

Generalized linear regression model:

logit(IsProf) ~ 1 + Institution + Gender + Age + Field + Score + Age^2 + Score^2

Estimated Coefficients:

Estimate SE tStat pValue

(Intercept) -21.909 1.5048 -14.559 5.0832e-48

Lincoln University -0.81268 0.32682 -2.4867 0.012894 Massey University -1.1813 0.22287 -5.3003 1.156e-07

University of Auckland -1.5287 0.23796 -6.4242 1.3254e-10

University of Canterbury -1.031 0.23829 -4.3265 1.5153e-05

University of Otago -0.91418 0.2186 -4.182 2.8892e-05

University of Waikato -0.85131 0.23862 -3.5676 0.00036031

Victoria University -1.611 0.24278 -6.6356 3.231e-11

Gender_Male 0.78864 0.1064 7.4121 1.243e-13

Age 0.44538 0.053789 8.2802 1.23e-16

Field_Science 0.65191 0.13714 4.7535 1.9994e-06

Field_Business 1.1107 0.15804 7.0278 2.0978e-12

Field_Engineering 0.52293 0.20612 2.537 0.01118

Field_Medicine 1.0003 0.17001 5.8837 4.0119e-09

Field_Education -0.42961 0.19999 -2.1481 0.031705

Score 0.01318 0.0029885 4.4103 1.0322e-05

Age^2 -0.0029567 0.0005084 -5.8156 6.0421e-09

Score^2 3.8569e-06 3.397e-06 1.1354 0.2562

4341 observations, 4323 error degrees of freedom

Chi^2-statistic vs. constant model: 2.84e+03, p-value = 0

GenderOddsRatio = 2.2004, AIC = 2.7972e+03

1. Modelling Salary

To predict salary, we tested all linear models with variables $Age$, $Institution$, $Field$, $Research score$ and $Gender$. Interactions between $Gender$ and all other terms were included, and both $Age$ and $Research score$ were squared. The best, and most parsimonious, model (see S3 Table for details of all models tested) as predicted with AIC was

$$Salary \sim Gender*\left( Field+ Age^{2} \right)+Research score^{2}+Institution.$$

Models that included interactions between Gender and all other terms showed a slightly lower AIC but as this difference was small (< 0.1) this more parsimonious model was used. Note that in contrast to the models for predicting the probability of being in the professoriate, research score squared was a useful predictor in this case.

To appreciate the model predictions, we used the same procedure to find an additional model to predict an individual’s research score over their lifetime. The best model, as predicted by AIC was

$$Research score \sim Gender*(Field+Age^{2})+Institution$$

Combining these two models allows us to predict the expected research score, and hence the expected salary of an individual working in a particular field at a particular institution.

Overall the predictive value of the best Research score model is low ($r^{2}=0.16$) showing the wide spread of research scores in the data. Salary predictions have a higher r-squared ($r^{2}=0.69$ for the best model). As expected Gender alone is the least useful of the single predictor variable models, as it is the only binary variable, but in both cases the best models without Gender had much lower AIC values than those with Gender. Overall, Gender is a significant variable when modelling Salary and Research score despite it adding only a small amount to the predictive power of the model.

Life time earnings (S1 Table) was the cumulative predicted annual salary for an individual with the predicted average research score from age 30 to 65. This was calculated for a man ($E_{M}$) and a woman ($E_{F}$) in each field. We also calculated the life time earnings for a woman with the expected male research score ($E_{FM}$). The lifetime gender pay gap was the difference between total expected male earnings and total expected female earnings, this difference include differences due to women’s expected lower research score. The proportion of the pay gap not attributable to research score differences was calculated as

$$\text{Pay gap not attributable to research difference}= 1-\frac{E_{M}-E_{FM}}{E_{M}-E_{F}}.$$

1. Research score and promotion

S2 Table shows the promotion chances of individuals in both the 2003 and 2012 dataset. Individuals were grouped by their 2003 rank and analysis was carried out on each group separately.

The score difference between men and women in each group was the gender coefficient (and associated p-value) from the linear regression model

$$Score2012 \sim Score2003+Age^{2}+Gender+Field.$$

The salary difference was the gender coefficient from the linear regression model

$$Salary2012 \sim Salary2003+Score2012+Gender+Age^{2}+Field.$$

The odds ratio for each of the promotions was the exponential of the gender coefficient (and associated p-value) of the logistic regression

$$Promoted to rank of interest\sim Gender+Age^{2}+Score2012+Field.$$

1. Exploring subject area

The six academic fields included in the analysis are a simplification of the 42 subject areas that are recorded in the PBRF exercise. Using a logistic regression model

$$P\left( Being AP or P \right)\sim Gender+SubjectArea+Research score+Age^{2}$$

gives men a significant advantage with a gender odds ratio of $2.11$ ($p={10}^{-12}$). Including interactions i.e.

$$P\left( Being AP or P \right)\sim Gender*SubjectArea+Research score+Age^{2}$$

the gender odds ratio for each subject area can be calculated. The average gender coefficient in the model is positive and still highly significant ($p\approx0.0015$), showing that again men are significantly more likely to be in the professoriate. We combine each specific subject area gender coefficient with the overall coefficient to give a subject area specific gender odds ratio (S5 Table, column I) for each of the 42 areas. In half the subject areas the Gender*Subject Area interaction is not significantly different from the overall coefficient (S5 Table, column J). However, the subject area specific odds ratio is less than 1, i.e. women are more likely to be in the professoriate, for only 9 of the 42 subject areas.

With the small numbers of individuals in some subject area/gender groupings these results may not be fully accurate for all subject areas. Overall they show that, even when Field is broken down into more specific groups, gender still has a significant effect in most subjects.

1. Modelling the future university

We set up a simple Leslie matrix model for the academic population at rank $R$ of each gender ($X=M$ or $F$) at time $t$, $N_{RX}(t)$. This gave eight separate classes (four ranks, two genders) of the form

$$N_{RX}\left( t+\Delta t \right)=N_{RX}\left( t \right)+ New hires+Promotions-Leaving.$$

The time step, $\Delta t$, was nine years, i.e. 2003 to 2012. First we calculated the number of individuals leaving the population of each class. The leaving rates were the number of individuals of that rank and gender in 2003 who had left by 2012, proportional to the number of individuals at that rank and gender (S4 Table).

After these individuals had been removed from each class, we then calculated the redistribution of individuals across ranks through the promotions process. The promotions transition rates were calculated using only individuals from 2003 who were still in the data in 2012. The probability of an individual at rank $i$ moving to rank $j$ was $P_{ij}$. Note that $\sum_{i} P_{ij}=1$ because it represents a transition matrix. A separate transition matrix was found for each gender.

After individuals had been redistributed across ranks, new hires were added to the population. As we were not concerned only with the proportion of individuals at each rank/gender, not with the total size of the academic population, the total number of new hires across all classes matched the total number of individuals, across all classes, who left. This kept the total population size stable.

The distribution of the new individuals across the rank and gender classes follows one of two models: current hiring practices (2B Fig) or equitable hiring (2C Fig). This allows the population in each class to change while the total population remains static.

The current hiring model allocates new hires by rank and gender following the distribution seen in the data. In other words, we take the total number of individuals who were present in 2012 but not in 2003 and calculate the fraction of these who were added to each rank/gender.

At the lecturer level, there is no significant difference in the gender split of new hires. Across all fields, approximately 15.6% of new hires are female lecturers and 14.6% are male lecturers. However at the professorial level, there is a clear difference. Overall 9.8% of new hires are male professors, in comparison under 2% of all new hires are female professors.

The equitable model splits new hires across the rank distribution seen in the data, but then allocates them equally to either gender. For example, 30.2% of new hires are at lecturer level and these are split evenly between male and female. This model allows for changes over time in the proportion of individuals at each rank, but isolates the effects of promotion and leaving rates from hiring decisions.

We also ran this model for each academic field (e.g. Arts, Science, etc.), using only datapoints from that field. Leaving rates varied widely across fields, usually between 40-60% of the population left in the 9 year period. On the whole, higher ranks had higher leaving rates.

Some fields with very small numbers of women had no leaving data available. For example, there was no leaving data were available for women in Engineering at the AP and P ranks, , because no-one at these ranks in 2003 had left by 2012. A t-test paired by gender of all rank/field leaving rates showed no significant difference in the rates for males and females ($p=0.43$). So the missing female rates were assumed to be the same as those for men. Also in engineering there was no data on women being promoted from the higher ranks. Because there was no significant gender difference at the higher ranks (S4 Table), we used the equivalent male data.

The new hires data also varies strongly with field. For example in Science, 12% of new hires are female lecturers, 18% are male lecturers, 13% are male professors and less than 1% are female professors (see S4 Table for details). However, in general the proportion of new hires that were female was not significantly different (Fisher’s exact test, $p>0.05$) from the proportion of females in the population (either 2003 or 2012) at any rank/field combination.

The model was run for the entire population and for each field separately. In all cases the population is the entire NZ academic population. An individual who moves from one university to another and rises in rank was recorded as a promotion, but is not considered to have left or be a new hire. The model reached a steady state after approximately 50 years. The initial condition for the model was the population sizes in 2012.

Table titles, (file names), and captions. Tables are available as excel spreadsheets.

S1 Table (S1_Table.xlsx)

Rank to salary conversion used for each institution. Taken from 2018 salaries in academic collective agreements available from [www.teu.ac.nz](http://www.teu.ac.nz).

S2 Table (S2_ Table.xlsx)

All possible logistic regression models to predict the probability of being in the professoriate (AP or P) in 2012 or 2003 separately. The table shows all regression models used, the gender coefficient (if included), associated p-value and corresponding odds ratio. Using the entire 2012 (or 2003) dataset the best fit model, as predicted by AIC, area under the receiver-operator curve or percentage of correct predictions, contains the gender variable. When a subset of the data is used (e.g. only A-grade researchers; only those under 50), or we predict the probability of being a full professor, gender is still a significant predictor variable in the best fit models.

S3 Table (S3_Table.xlsx)

All possible linear regression models to predict an individual’s salary and PBRF score in 2012. The table shows all regression models used, the gender coefficient (if included), associated p-value and corresponding odds ratio. Note that we used interactions between gender and other terms in these models, rendering the gender coefficient less explanatory in this case. The sample is the same as that in Table S2. The sample size of men and women is given. For the salary model, the top four models showed almost no difference by AIC so the most parsimonious, i.e. the model with the least interactions, was chosen. Choosing one of the other models gave a slight quantitative change to Table 2 but did not change the overall results.

S4 Table (S4_Table.xlsx)

Promotion rates, hiring probabilities, and leaving rates for each field, as used in the Leslie matrix transition model.

S5 Table (S5_Table.xlsx)

The probability of being in the professoriate using all 42 subject areas.

**References for Supporting Information**

1. New Zealand Tertiary Education Commission. Performance-Based Research Fund - Quality Evaluation Guidelines 2012. Wellington, New Zealand; 2013.

2. New Zealand Tertiary Education Commission. Performance-Based Research Fund. 2019 [cited 2019 Apr 17]. Available from: https://www.tec.govt.nz/funding/funding-and-performance/funding/fund-finder/performance-based-research-fund/

3. Buckle RA, Creedy J. The Impact on Research Quality of Performance-Based Funding: The Case of New Zealand’s PBRF Scheme. Agenda - A J Policy Anal Reform. 2018;25(1):25–48.

4. Buckle RA, Creedy J. The evolution of research quality in New Zealand universities as measured by the performance-based research fund process. New Zeal Econ Pap. 2018;53(2):144–65.

5. Buckle RA, Creedy J. An evaluation of metrics used by the Performance-based Research Fund process in New Zealand. New Zeal Econ Pap. 2018;53(2):270–87.
