# Supplementary figures and images for: Research performance and age explain less than half of the gender pay gap in New Zealand universities
Source: PLoS One. 2020 Jan 22;15(1):e0226392. doi: 10.1371/journal.pone.0226392 (PMC6975525; doi:10.1371/journal.pone.0226392)

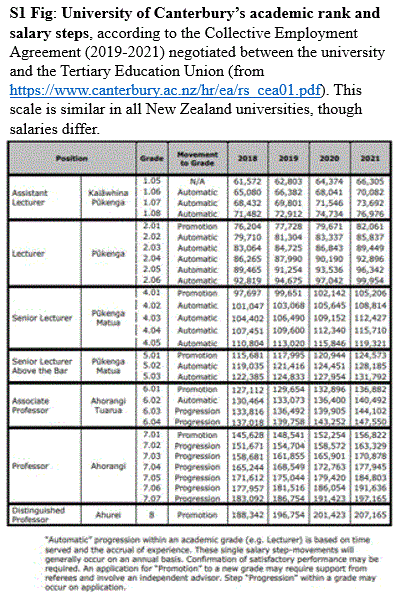

Supplement: S1 Fig — This scale is similar in all New Zealand universities, though salaries differ. (TIF) [file pone.0226392.s002.tif]

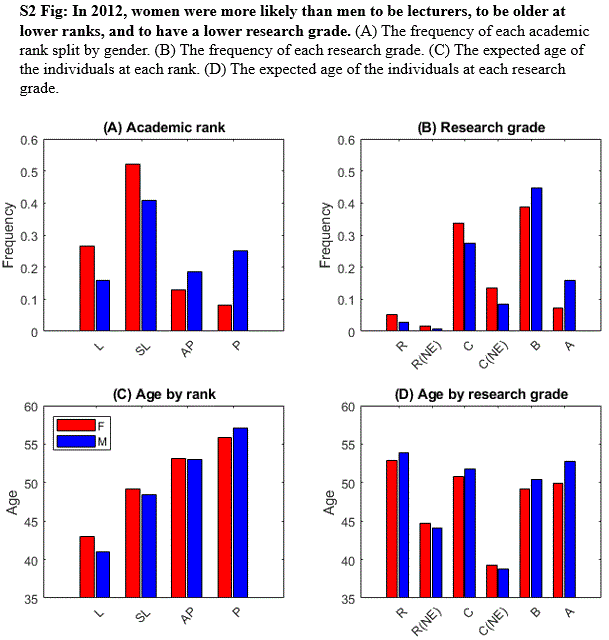

Supplement: S2 Fig — (A) The frequency of each academic rank split by gender. (B) The frequency of each research grade. (C) The expected age of the individuals at each rank. (D) The expected age of the individuals at each research grade. (TIF) [file pone.0226392.s003.tif]
